# Estimating cumulative pathway effects on risk for age-related macular degeneration using mixed linear models

**DOI:** 10.1186/s12859-015-0760-4

**Published:** 2015-10-14

**Authors:** Jacob B. Hall, Jessica N. Cooke Bailey, Joshua D. Hoffman, Margaret A. Pericak-Vance, William K. Scott, Jaclyn L. Kovach, Stephen G. Schwartz, Anita Agarwal, Milam A. Brantley, Jonathan L. Haines, William S. Bush

**Affiliations:** Graduate Program in Human Genetics, Vanderbilt University, Nashville, TN USA; Department of Epidemiology and Biostatistics, Case Western Reserve University, Cleveland, OH USA; John P. Hussman Institute for Human Genomics, University of Miami Miller School of Medicine, Miami, FL USA; Department of Ophthalmology at Bascom Palmer Eye Institute, University of Miami Miller School of Medicine, Miami, FL USA; Department of Ophthalmology and Visual Sciences, Vanderbilt University, Nashville, TN USA; Institute for Computational Biology, Department of Epidemiology and Biostatistics, Case Western Reserve University, Cleveland, OH USA

**Keywords:** Age-related macular degeneration (AMD), Heritability, Pathway analysis, Mixed linear model (MLM), Proportion of variance explained (PVE)

## Abstract

**Background:**

Age-related macular degeneration (AMD) is the leading cause of irreversible visual loss in the elderly in developed countries and typically affects more than 10 % of individuals over age 80. AMD has a large genetic component, with heritability estimated to be between 45 % and 70 %. Numerous variants have been identified and implicate various molecular mechanisms and pathways for AMD pathogenesis but those variants only explain a portion of AMD’s heritability. The goal of our study was to estimate the cumulative genetic contribution of common variants on AMD risk for multiple pathways related to the etiology of AMD, including angiogenesis, antioxidant activity, apoptotic signaling, complement activation, inflammatory response, response to nicotine, oxidative phosphorylation, and the tricarboxylic acid cycle. While these mechanisms have been associated with AMD in literature, the overall extent of the contribution to AMD risk for each is unknown.

**Methods:**

In a case–control dataset with 1,813 individuals genotyped for over 600,000 SNPs we used Genome-wide Complex Trait Analysis (GCTA) to estimate the proportion of AMD risk explained by SNPs in genes associated with each pathway. SNPs within a 50 kb region flanking each gene were also assessed, as well as more distant, putatively regulatory SNPs, based on DNaseI hypersensitivity data from ocular tissue in the ENCODE project.

**Results:**

We found that 19 previously associated AMD risk SNPs contributed to 13.3 % of the risk for AMD in our dataset, while the remaining genotyped SNPs contributed to 36.7 % of AMD risk. Adjusting for the 19 risk SNPs, the complement activation and inflammatory response pathways still explained a statistically significant proportion of additional risk for AMD (9.8 % and 17.9 %, respectively), with other pathways showing no significant effects (0.3 % – 4.4 %).

**Discussion:**

Our results show that SNPs associated with complement activation and inflammation significantly contribute to AMD risk, separately from the risk explained by the 19 known risk SNPs. We found that SNPs within 50 kb regions flanking genes explained additional risk beyond genic SNPs, suggesting a potential regulatory role, but that more distant SNPs explained less than 0.5 % additional risk for each pathway.

**Conclusions:**

From these analyses we find that the impact of complement SNPs on risk for AMD extends beyond the established genome-wide significant SNPs.

**Electronic supplementary material:**

The online version of this article (doi:10.1186/s12859-015-0760-4) contains supplementary material, which is available to authorized users.

## Background

In developed countries the number one cause of irreversible visual loss in the elderly is age-related macular degeneration (AMD) [1, 2]. AMD is a progressive neurodegenerative disease leading to loss of central vision through dysfunction or death of photoreceptors in the macula. This loss of vision often impacts quality of life, depending on the severity and speed of disease progression in a given individual, with geographic atrophy (GA; an advanced form of non-neovascular or “dry” AMD) typically progressing much more slowly and with less severe symptoms than choroidal neovascularization (CNV; neovascular “wet” AMD). Because the average age of most populations is increasing, the prevalence of AMD is also expected to increase worldwide to approximately 196 million by 2020 [2].

AMD has long been known to have a genetic component [3]; a twin study found that 46 % to 71 % of the variation in the overall severity of AMD was explained by additive genetic effects [4]. Variation in the Complement Factor H (*CFH*) gene, was associated with AMD in 2005 [5–7], and several loci have since been associated [8]. Certain genetic variants contribute more to risk for one subtype of AMD than another—for example, *CFH* risk alleles are preferentially associated with risk for GA and *ARMS2* risk alleles are preferentially associated with risk for CNV [8–10]. Additionally, AMD prevalence differs by ethnic background, with European-descent individuals having higher prevalence rates than African-decent, Asian-descent, and Hispanic individuals [2]. Many advancements have been made toward understanding AMD pathogenesis, yet it is far from being fully elucidated. No cure for AMD exists and most treatment methods aim to prevent or slow disease progression [11].

Multiple mechanisms have been proposed as having a role in AMD pathogenesis. A recent review of AMD by Fritsche et al. describes many risk factors and mechanisms [11]. The discovery of the association between the Complement Factor H gene and AMD led to further associations between other genes related to *complement activation* [12]. *Inflammation* is highly related to complement activation, and can lead to apoptosis of retinal pigment epithelial (RPE) cells and photoreceptors [13]. *Apoptosis* is thought to be more deeply associated with AMD; using terminal deoxynucleotidyl transferase dUTP nick end-labeling (TUNEL), Dunaief et al. found that RPE cells, photoreceptors, and inner nuclear layer cells can die through apoptosis during AMD progression [14]. The Age-Related Eye Disease Study (AREDS) showed that antioxidant vitamin supplements were able to slow AMD progression [15], implicating *antioxidant* mechanisms as candidates in disease progression. These include intermediates of the *tricarboxylic acid cycle* (TCA cycle), which can alter the effectiveness of zeaxanthin (a component of AREDS2 supplements) [16]. Zhao and Vollrath showed that when mitochondria in RPE were ablated in mice, the lack of oxidative *phosphorylation* (OxPhos) in the RPE led to photoreceptor degeneration [17]. *Angiogenesis* is known to play a significant role in choroidal neovascularization (CNV), and anti-VEGF treatments, which aim to inhibit angiogenesis, are used as treatment for wet AMD [18]. Finally, smoking is a well-known risk factor for AMD [19], and thus *nicotine metabolism* may plausibly play a role in AMD pathogenesis. While there is substantial evidence that complement activation plays a major role in AMD, the genetic mechanisms involved in other mechanisms are less established.

Genetic variants with large effect sizes, several of which are localized to complement system genes, have been repeatedly associated with AMD [5, 6, 8, 12, 20]. However, AMD-associated SNPs that reach genome-wide significance only account for a portion of the known heritability [8]. SNPs with smaller effects likely contribute cumulatively to an additional portion of the heritability. While overall heritability estimates of AMD are known, the estimated contribution to heritability, separately, for many AMD-related pathways is unknown. Existing genetic pathway analysis methods typically annotate SNP associations using databases such as the Gene Ontology (GO) [21], Ingenuity Pathway Analysis (IPA) [22], the Kyoto Encyclopedia of Genes and Genomes (KEGG) [23], or Reactome [24]. These methods then utilize analytical approaches, such as Gene Relationships Across Implicated Loci (GRAIL), Gene Set Analysis (GSA), and Pathway Analysis by Randomization Incorporating Structure (PARIS), to determine the significance of pathways, usually using gene or SNP *p*-values or genotype data to calculate a rank-based pathway statistic [25]. These methods, however, do not provide a scaled measure of the effect and thus do not offer estimates of heritability or the proportion of overall disease risk explained by an entire pathway. In this study, using a case–control AMD cohort, we estimate the significance and proportion of risk explained by additive genetic effects within specific AMD-related pathways in order to prioritize them for future molecular and epidemiological studies.

## Methods

### Dataset summary

Subjects in this study (Table 1) were recruited from the Duke University Eye Center (DUEC), the Vanderbilt Eye Institute (VEI), and the Bascom Palmer Eye Institute (BPEI) at the University of Miami Miller School of Medicine starting in 1995, 1999, and 2007, respectively. Individuals were recruited through retinal clinics, mostly via referrals for possible AMD; recruitment was performed under research protocols approved by the Duke University School of Medicine Institutional Review Board, the Vanderbilt University Institutional Review Board, and the University of Miami Institutional Review Boards, and written informed consent was obtained from all participants. Original recruitment was performed for a previous study of AMD [26] and permission to use the dataset for this study was obtained. Controls were recruited either as friends or spouses of cases or through regular eye exams. Examination, imaging, and grading were performed prior to the start of this analysis. All subjects were examined by a retina specialist using slit-lamp biomicroscopy and dilated fundus examination, including indirect ophthalmoscopy. Additionally, fundus imaging was analyzed to confirm case status. For consistency between sites, images were scored by a retina specialist using a modified grading system based on the Age-Related Eye Disease Study (AREDS) [27]. The grading system was used to score individuals on a scale between 1 and 5. Subjects with grades 1 and 2 were considered controls and subjects with grades 3 through 5 were considered cases, with grade 3 representing early AMD (non-neovascular) and grades 4 and 5 representing late AMD (GA and CNV, respectively). Both eyes were scored and an individual’s overall grade was determined using the eye with the higher grade.

**Table 1:** Study population characteristics

Cohort	Age^a^ (SD)	Males (%)	Smokers (%)
Primary subset - 1,813 (100 %)	75.1 (8.4)	713 (39.3)	—
Cases - 1,145 (63.2 %)	77.6 (7.9)	415 (36.2)	—
Controls - 668 (36.8 %)	70.9 (7.7)	298 (44.6)	—
Smoking subset - 1,358 (100 %)	75.0 (8.2)	560 (41.2)	790 (58.2)
Cases - 850 (62.6 %)	77.3 (7.7)	323 (38.0)	516 (60.7)
Controls - 508 (37.4 %)	71.2 (7.6)	237 (46.7)	274 (53.9)

^a^Mean age in years

Primary cohort contains all individuals after QC measures were applied

Smoking subset cohort excludes individuals with unknown smoking status

### Genotyping and quality control

Three genotyping platforms were used: the Affymetrix 1M array (906,600 SNPs), a custom Sequenom array (84 SNPs), and custom TaqMan assays (4 SNPs). The Sequenom array was designed to interrogate potential AMD-related SNPs, while the TaqMan assays were used later to validate SNPs that performed poorly on the Sequenom array. SNP quality control (QC) was performed separately for Affymetrix SNPs and for merged Sequenom/TaqMan SNPs and was applied simultaneously to cases and controls. For the Affymetrix genotyping chip, 38,443 non-autosomal SNPs were removed, 102,735 SNPs with genotyping efficiency < 95 % were removed, 104,695 SNPs with a minor allele frequency (MAF) < 1 % were removed, 1,475 SNPs with Hardy-Weinberg Equilibrium (HWE) *p*-values < 1 × 10^−6^ were removed, 121 SNPs not able to be converted from genome build 36 to 37 using liftOver [28] were removed, and 25 Affymetrix SNPs that were present in post-QC Sequenom/TaqMan SNPs were removed, resulting in 659,106 post-QC Affymetrix SNPs. QC procedures were applied to 88 merged Sequenom/TaqMan SNPs for 1,911 individuals that also had Affymetrix data. Forty-five individuals were removed that had genotyping efficiency < 90 %, leaving 1,866 individuals. For the merged data, 4 non-autosomal SNPs were removed, no SNPs had a genotyping efficiency < 95 %, 7 SNPs with a MAF < 1 % were removed, and 2 SNPs with a HWE *p*-value < 1 × 10^−6^ were removed, leaving 75 SNPs for analysis. All merged genotype platforms resulted in a total of 659,181 SNPs for analysis.

All 1,967 individuals in our dataset were observer-reported to be white (European American), however we performed principal components analysis using 71 ancestry informative markers, seeding with six distinct HapMap phase 3, release 3 populations [29], to confirm genetic ancestry (Additional file 1: Figure S1). Twelve individuals with non-European American genetic ancestry were removed to avoid potential confounding by population stratification, including eleven with African American genetic ancestry and one with Asian genetic ancestry (Additional file 1: Figure S1). Additionally, five individuals were removed that had genotyping efficiency less than 90 %, based on Affymetrix genotype data, 84 individuals were removed that did not have available Sequenom/TaqMan genotype data, and 53 individuals were removed that did not have age recorded at time of examination, leaving 1,813 individuals for analysis (1,145 cases and 668 controls). Finally, some of our analyses required individuals to have known smoking status, with individuals considered to be smokers if they had smoked 100 or more cigarettes in their life; 455 individuals did not have available smoking status information, leaving 1,358 individuals for smoking status adjusted analyses (Table 1). The distribution of age was similar between cases and controls (Additional file 1: Figure S2).

### Pathway selection and curation

For this study our goal was to determine the overall contribution of several pathways on AMD risk, to both confirm the importance of known mechanisms (e.g. complement activation) and to determine if some biological mechanisms contribute to cumulative AMD risk without harboring individual genome-wide significant, large-effect genetic variants. Based on an extensive literature search (as summarized in the third paragraph of the background section) and advice from AMD experts, we chose eight mechanisms ranging from having plausible to extremely well-known AMD relation to test as pathways (Table 2) in our analysis.

**Table 2:** Gene ontology terms used to define pathways

GO Term	GO ID	# Genes	Reference
Angiogenesis	GO:0001525	379	PMID: 23642783
Antioxidant activity	GO:0016209	69	PMID: 23645227
Apoptotic signaling	GO:0097190	1,635	PMID: 12427055
Complement activation	GO:0006956	187	PMID: 20711704
Inflammatory response	GO:0006954	534	PMID: 17021323
Response to nicotine	GO:0035094	31	PMID: 8827967
Oxidative phosphorylation	GO:0006119	78	PMID: 21483039
Tricarboxylic acid cycle	GO:0006099	33	PMID: 14962143

The Gene Ontology (GO) [21] is a database of hierarchical gene relationships. To objectively determine genes related to each of the eight selected pathways we selected appropriate GO terms corresponding to each pathway and (Table 2) extracted all associated genes (Additional file 2) falling under the hierarchy of that GO term using the November 2013 release of the GO database. Because GO is hierarchical, containing parent–child-type relationships, we included all descendants of the selected GO terms so as to not omit directly related genes. For each assigned gene, we tested three partitioned regions to represent the effect of that gene (Additional file 1: Figure S3), including (1) SNPs within Ensembl-defined gene boundaries, (2) SNPs within 50 kb flanking each gene boundary (to capture *cis*-regulatory SNPs), and (3) SNPs within 50 kb and 250 kb flanking each gene that also lie within open chromatin regions based on ENCODE DNaseI hypersensitivity analyses from human retinal pigment epithelial cells (hRPEpiC) [30].

### Mixed linear model analysis

To estimate the proportion of AMD risk explained by each pathway, we used Genome-wide Complex Trait Analysis (GCTA) [31] to fit genetic relationship matrices (GRMs) in mixed linear models (MLMs) via the restricted maximum likelihood (REML) method. GRMs contain information about the genetic relationship (by additive genetic effects) between all pairs of individuals in a dataset, and can be calculated separately for different combinations or subsets of SNPs. GCTA performs likelihood ratio tests (LRTs), comparing full and reduced models to determine the significance of a given genetic variance component, where the reduced model is created by dropping the genetic variance component (GRM of interest) from the full model. Whereas with continuous, quantitative traits, the proportion of phenotypic variance explained (PVE) by specified SNPs is estimated, for case–control studies a liability threshold model is implemented to estimate the proportion of risk explained (PRE).

For many analyses we tested three different REML algorithms—average information (AI), Fisher-scoring, and expectation maximization (EM); here, we will only show results using the EM algorithm, which was computationally slower but provided slightly more reliable model fitting. For all analyses we included age, sex, and the first two principal components as covariates. For case–control analyses, GCTA by default uses disease prevalence rates observed within a dataset; however, it is recommended to use prevalence rates from general populations based on literature. We used a prevalence rate (Additional file 1: Table S1) of 5.07 %, calculated by weighting all individuals in our dataset with U.S. prevalence rates, stratified by age [32]. The proportion of risk explained is then transformed from the observed scale to the specified prevalence scale. Linkage disequilibrium (LD) has a minimal effect on estimates from GCTA, with studies showing that cumulative estimates are stable and not necessarily over-inflated because both influential and non-influential SNPs in LD are considered and therefore possible confounding effects are neutralized [33, 34]. To explore potential LD effects within our study, we perform additional analyses on SNP sets pruned using LD.

We estimated the overall proportion of risk for AMD explained, as well as the proportion of risk explained by each pathway for various gene regions and exclusion criteria (Additional file 1: Figure S3). We explored effects of LD, SNP overlap between pathways, smoking status, and stratification by AMD subtype on the proportion of AMD risk explained, either cumulatively or by pathway. Again, all analyses were adjusted for age, sex, and the first two principal components, using an adjusted prevalence rate of 5.07 %. The following are more detailed methods for each specific analysis.

### Genome-wide AMD risk explained

The first analysis we performed was to assess the overall proportion of AMD risk explained by all available genotyped SNPs in our dataset (often referred to as “chip heritability”). One GRM was created for all 659,181 SNPs and was included in a mixed linear model analysis using GCTA, adjusting for the covariates described previously.

### Known risk SNPs

A recent meta-analysis [8] of AMD describes 19 genome-wide significant AMD risk SNPs (Additional file 1: Table S2). To determine the effect that those 19 known SNPs have in our dataset we created a GRM consisting of just those 19 SNPs, referred to as the risk GRM, and a GRM for all other SNPs (659,162), referred to here as the remainder GRM. Additionally, we created risk GRMs that included 5 kb and 50 kb flanking (and including) the 19 known risk SNPs, resulting in a total of 83 and 566 risk SNPs, respectively, with the remainder GRM being all SNPs minus the given risk subset.

### Risk explained by pathways

To estimate the effect of the eight selected AMD-related pathways, two GRMs were generated, unless otherwise specified, for each analysis of each pathway. Pathway GRMs consist of SNPs being assessed for a respective pathway and remainder GRMs contain all other SNPs being considered that are not in the respective pathway GRM and that are not excluded. Many pathways have overlapping genes and thus effects from all pathways could not be estimated in a single mixed linear model. We assessed the effect for several gene regions (Additional file 1: Figure S3), starting with just genic SNPs, then subsequently adding SNPs within 50 kb flanking each gene, and then SNPs in open chromatin regions within 50 kb to 250 kb flanking each gene, based on the ENCODE DNaseI hypersensitivity data from human retinal pigment epithelial cells (hRPEpiC). Additionally, for each pathway we performed analyses excluding 5 kb risk regions around and encompassing each of the 19 known risk SNPs from the regions including genic SNPs, SNPs within 50 kb flanking, and more distant SNPs in open chromatin regions. When known risk regions were excluded from a pathway GRM, they were not included in the remainder GRM but were rather excluded entirely, so as to determine cumulative, additional risk explained by pathways. Finally, we calculated the risk explained for each pathway adjusting for the number of SNPs in each pathway to ensure that the amount of risk explained was not simply due to the number of SNPs included in a given pathway.

### Gene overlap

For this study it was not feasible to allow all pathways to have unique, non-overlapping gene sets. Thus, we tested the overlap between all pairs of pathways to determine whether risk explained was unique to certain pathways or shared between pathways due to sharing of common genes. For each overlapping pathway we created a GRM using overlapping SNPs and a GRM using non-overlapping SNPs, based on genic SNPs and 50 kb flanking.

### Linkage disequilibrium near known risk SNPs

While we assessed excluding risk SNPs and 5 kb flanking those risk SNPs from each pathway, SNPs in more distant LD with those risk SNPs could influence the calculation of pathway GRMs and inflate estimates of the proportion of risk explained. Thus, we used LD information from CEPH individuals in HapMap phase II to exclude all SNPs in LD with the 19 known risk SNPs. We used exclusion criteria of R^2^ ≥ 0.10, 0.05, and 0.01, much more strict than the typically used R^2^ cutoff of ≥ 0.80, therefore removing SNPs with even minimal LD to known risk SNPs. Each SNP had LD information for other SNPs within a 500 kb flanking region. To be even more conservative we also excluded 1 MB regions flanking each risk SNP. For each threshold we created a remainder GRM for all SNPs minus any matching the exclusion criteria. Results were compared to previous estimates of AMD risk explained by known risk SNPs and all other genotyped SNPs to estimate risk explained due to LD near risk SNPs. Each analysis included a risk GRM and a remainder GRM.

### Effect of smoking status

Smoking is a major risk factor for the development of AMD [35], so we also ran additional analyses for each pathway, including smoking status as a covariate, to detect any differences in significance or amount of risk explained per pathway, when adjusting for smoking. Genic SNPs plus 50 kb flanking were used to compare effects. Of the 1,813 individuals used in this study 455 did not have available smoking status.

### Stratification by AMD subtype

We ran analyses stratifying by AMD subtype to confirm that our dataset exhibits no AMD-subtype effect, especially considering that some pathways analyzed are by definition more related to a particular AMD subtype (e.g. angiogenesis is highly related to neovascular AMD). For these analyses we excluded individuals with early AMD (grade 3) and considered only controls versus grade 4 (CNV) and controls versus grade 5 (CNV in at least one eye). We tested genic SNPs plus 50 kb flanking plus open chromatin for both subtypes of AMD for each pathway.

## Results and Discussion

### Genome-wide AMD risk explained

In our first analysis we used all 659,181 genotyped SNPs that passed QC to estimate the heritability of AMD in our dataset. We found that 61.5 % (*p*-value = 3.4 × 10^−5^; S.E. = 16.9 %) of the risk for AMD in our dataset was explained by those SNPs, in range of known AMD heritability estimates. This confirmatory step helps validate subsequent pathway analyses in this study, showing that there is substantial variation in our dataset that impacts AMD risk. When assessed separately, the 19 previously associated AMD risk SNPs explained 13.30 % of the risk for AMD in our dataset (*p* = 1.35 × 10^−61^) while other genotyped SNPs explained 36.72 % of the risk. Regions flanking the risk SNPs were also considered in separate analyses and explained a total of 15.37 % (*p* = 1.59^−53^) when 5 kb flanking the risk SNPs were included, and 16.33 % (*p* = 8.24^−44^) when 50 kb flanking regions were included. From this we see that known risk SNPs explain only a portion of the overall risk estimate, indicating that additional lower-effect SNPs may influence disease risk.

### Risk explained by pathways

**Fig. 1:**
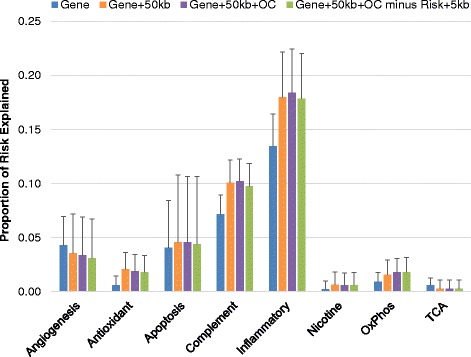
Risk explained by each pathway, by partitioning strategy. Each bar represents the proportion of risk explained from a fitted mixed linear model using SNPs selected for each pathway for four different partitioning strategies. Error bars represent standard error (SE)

We first assessed the effect of each pathway for three different gene region inclusion criteria without excluding any known risk SNPs (Fig. 1). The complement and inflammatory pathways explained between approximately 10 % (*p* < 1 × 10^−25^) and 17 % (*p* < 1 × 10^−7^), respectively, of the risk for AMD, while the angiogenesis and apoptotic signaling pathways explained nearly 5 % of the risk (non-significant), and the antioxidant, nicotine, oxidative phosphorylation, and tricarboxylic acid cycle pathways explained approximately 2 % of the risk or less (non-significant). In general, we observed that inclusion of SNPs within 50 kb flanking pathway genes typically increased the amount of risk explained, while additional inclusion of more distant SNPs in open chromatin regions did not explain a great deal more risk, suggesting that local regulatory SNPs indeed modulate risk.

We also assessed each pathway, excluding known risk SNPs and 5 kb flanking (referred to as risk regions) from regions including genic SNPs plus 50 kb flanking plus open chromatin SNPs, to better estimate novel risk explained by each pathway (Fig. 1, green bars). We observed very little reduction in the amount of risk explained by each pathway when the risk regions were removed. The response to nicotine, oxidative phosphorylation, and tricarboxylic acid cycle pathways contained no SNPs within risk regions, while other pathways contained at most 10 SNPs within risk regions to be removed, indicating that risk explained by each pathway is in addition to the amount of risk explained by the 19 known risk SNPs.

**Fig. 2:**
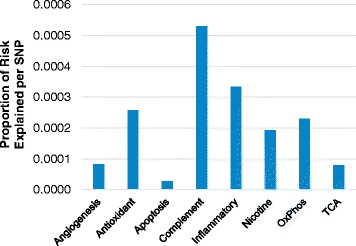
Average risk explained per SNP by pathway. Each bar represents the proportion of risk explained divided by the number of SNPs per pathway. In this analysis, risk SNPs plus 5 kb regions were excluded

Notably, the number of genes and SNPs differs significantly over the pathways we targeted. When we adjusted the proportion of risk explained from each pathway by the number of SNPs contained within each pathway, we observed results consistent with known genetic contributors to AMD (Fig. 2). Unsurprisingly, after adjusting for the number of SNPs in each pathway, the complement pathway explains the highest amount of risk per SNP. The antioxidant, nicotine, and oxidative phosphorylation pathways, which each explain less 2 % of the risk for AMD, have similar levels of per-SNP effects (about 0.02 %), on the same order of magnitude as the complement pathway (0.05 %) and inflammatory pathway (0.03 %). Overall, we see very little cumulative effect of SNPs outside the complement and inflammatory pathways, but identify additional risk from complement and inflammatory mechanisms, due in part to variation within the flanking regions of these genes that is likely to be regulatory.

### Gene overlap

**Fig. 3:**
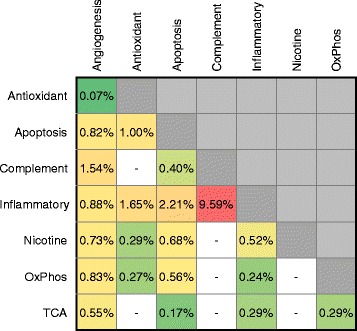
Risk explained by overlapping SNPs between pathway pairs. Values represent the proportion of risk explained for SNPs contained in each pathway overlap. Pathway pairs with no overlapping SNPs shown as white boxes. Pathway pairs with less risk explained by overlap shown as green, fading to red for pathway pairs with more risk explained by overlap. Overlap was calculated using gene plus 50 kb regions

**Fig. 4:**
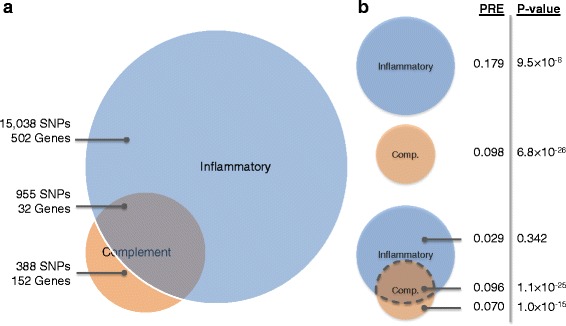
Overlap between complement and inflammatory pathways. **a** Venn diagram of SNP and gene overlap between the complement and inflammatory pathways. **b** *P*-values and the proportion of risk explained (PRE) by complement and inflammatory pathways, separately and for overlapping regions. Overlapping SNPs were determined using regions including genic SNPs plus 50 kb flanking regions

The pathways we selected to study for association to AMD risk were not all completely unrelated. For example, inflammation, apoptotic signaling, and angiogenesis are all biologically related and also have SNP overlap between pathways (Additional file 1: Figure S4). We estimated the proportion of risk explained due to SNPs overlapping between pathways for each pathway pair where overlap was present and found that the overlap between most pathway pairs accounted for between 0.07 % and 2.21 % of the risk for AMD explained (Fig. 3). The SNPs overlapping between the complement and inflammatory pathways, however, explained 9.59 % of the risk for AMD; taking a closer look at SNPs shared provides a better understanding of the risk explained by the two pathways (Fig. 4). Of the 1,343 SNPs in the complement pathway, 955 were also in the inflammatory pathway. The 15,038 SNPs unique to the inflammatory pathway, however, only explained 2.9 % of the risk for AMD—a non-statistically significant amount. From this we observe that while the inflammatory pathway, at first glance, appears to explain more risk than the complement pathway, in reality, a large amount of the risk, but not all, is due to genes shared between the complement activation pathway.

### Linkage disequilibrium near known risk SNPs

In order to ensure that SNPs near the 19 known risk SNPs (Additional file 1: Table S2) were not overinflating estimates of risk explained, we used LD information around the risk SNPs to exclude SNPs in LD and measure any changes in overall, genome-wide estimates of AMD risk explained (Additional file 1: Table S3). As mentioned previously, the 19 risk SNPs alone explained 13.3 % of risk for AMD while all other SNPs (included in a remainder GRM) explained 36.7 % of the risk for AMD. Exclusion of SNPs using the threshold of R^2^ ≥ 0.01 only reduced the risk explained by 1.6 %, to 35.1 %. In an even more conservative case, we excluded 1 MB flanking each side of the risk SNPs, regardless of LD, resulting in a reduction in risk explained of 5.4 %, to 31.3 %—unsurprising given the number of total SNPs excluded. Based on this we can assume that LD between risk SNPs and pathways SNPs would not confound estimates of AMD risk explained.

### Effect of smoking status

Smoking is a major risk factor for AMD; therefore, we assessed the impact of smoking status as a covariate in a sub-analysis of these data in samples where smoking status was available. After adjusting for smoking, the proportion of risk explained by each pathway did not change considerably (Additional file 1: Figure S6). In fact, after adjustment, the angiogenesis, complement, and inflammatory pathways actually explained slightly more risk for AMD. All pathways exhibited little change and we conclude that adjusting for smoking status does not modulate the cumulative effect of SNPs within any of the targeted pathways.

### Stratification by AMD subtype

Finally, we compared the effects on AMD risk, stratified by AMD subtype, for all pathways (Additional file 1: Figure S7). There were 668 controls, 1,145 total AMD cases, 113 cases with GA (grade 4; advanced dry AMD), 667 cases with CNV (grade 5 in at least one eye; wet AMD, and 365 cases with grade 3 or an unrecorded grade. We hypothesized that, because of the strong biological correlation between wet AMD and the angiogenesis pathway, a significant proportion of risk explained by the angiogenesis pathway would be observed when comparing CNV cases to controls. However that was not the case; we observe slightly more risk explained by the angiogenesis pathway (and most other pathways) when comparing cases with GA to controls. We observe an unusual peak of risk explained by the apoptosis pathway when comparing GA versus controls, which is intriguing given possible associations with GA and apoptosis in literature. However, this signal may be an artifact of the more limited power within our GA subset.

## Conclusions

In our analyses, we both confirm existing knowledge of AMD genetics and provide new, additional information on putative disease-associated pathways influencing risk for AMD. Our results show that SNPs in genes (and within 50 kb flanking) associated with complement activation and inflammation significantly contribute to AMD risk, separately from the risk explained by 19 known risk SNPs. We note, however, that the complement and inflammatory pathways are not discrete; we found that a large proportion of risk explained by the inflammatory pathway is due to overlap with complement activation genes. Other mechanisms thought to be involved in AMD pathogenesis do not appear to greatly influence disease risk through the cumulative action of common genetic variants. We also observe that while smoking is a known risk factor for AMD, inclusion as a model covariate does not significantly affect risk estimates from pathways. Overall, we show genes that interplay between the complement and inflammatory pathways explain additional risk, apart from the known, large-effect AMD risk SNPs, and that some portion of these are localized to the 50 kb flanking regions, indicating a regulatory role. As such, further targeted genomic or molecular studies may wish to prioritize additional loci within the complement pathway over other proposed disease mechanisms.

### Availability of supporting data

Data used in this study is being deposited into dbGaP. Please contact the corresponding author to request supporting data.

## Additional files

Additional file 1:
**Supplementary Document.** This PDF document provides additional figures and tables not included in the paper, including information on principal components analysis, age distributions, details of gene regions analyzed, details of SNP overlap between pathways, analyses adjusting for smoking status, analyses stratifying by AMD subtype, details of calculating expected population prevalence rate, information on the 19 known risk SNPs, analyses of linkage disequilibrium effects, and pathway SNP counts. (DOCX 766 kb) Additional file 2:
**Pathway Gene Lists.** This Excel file contains lists of all genes contained within each pathway assessed—one column per pathway. (XLSX 45 kb)

## Abbreviations

AI: Average Information
AMD: Age-related Macular Degeneration
AREDS: Age-Related Eye Disease Study
ARMS2: Age-Related Maculopathy Susceptibility 2 Gene
BPEI: Bascom Palmer Eye Institute
CFH: Complement Factor H Gene
CNV: Copy Number Variant
DUEC: Duke University Eye Center
EM: Expectation Maximization
GA: Geographic Atrophy
GCTA: Genome-wide Complex Trait Analysis
GO: The Gene Ontology
GRAIL: Gene Relationship Across Implicated Loci
GRM: Genetic Relationship Matrix
GSA: Gene Set Analysis
hRPEpiC: Human Retinal Pigment Epithelial Cells
HWE: Hardy Weinberg Equilibrium
IPA: Ingenuity Pathway Analysis
KEGG: The Kyoto Encyclopedia of Genes and Genomes
LD: Linkage Disequilibrium
LRT: Likelihood Ratio Test
MAF: Minor Allele Frequency
MLM: Mixed Linear Model
OxPhos: Oxidative Phosphorylation
PARIS: Pathway Analysis by Randomization Incorporating Structure
PRE: Proportion of Risk Explained
PVE: Proportion of Variance Explained
QC: Quality Control
REML: Restricted Maximum Likelihood
RPE: Retinal Pigment Epithelium
TCA: Tricarboxylic Acid
TUNEL: Terminal deoxynucleotidyl transferase dUTP Nick End-Labeling
VEI: Vanderbilt Eye Institute
