# Supplementary material for: Estimating cumulative pathway effects on risk for age-related macular degeneration using mixed linear models
Source: BMC Bioinformatics. 2015 Oct 14;16:329. doi: 10.1186/s12859-015-0760-4 (PMC4606903; doi:10.1186/s12859-015-0760-4)
Supplement: Additional file 1: — Supplementary Document. This PDF document provides additional figures and tables not included in the paper, including information on principal components analysis, age distributions, details of gene regions analyzed, details of SNP overlap between pathways, analyses adjusting for smoking status, analyses stratifying by AMD subtype, details of calculating expected population prevalence rate, information on the 19 known risk SNPs, analyses of linkage disequilibrium effects, and pathway SNP counts. (DOCX 766 kb) [file 12859_2015_760_MOESM1_ESM.docx]

# Estimating cumulative pathway effects on risk for

# age-related macular degeneration using mixed linear models

Jacob B Hall, Jessica N Cooke Bailey, Joshua D Hoffman, Margaret A Pericak-Vance, William K Scott, Jaclyn L Kovach, Stephen G Schwartz, Anita Agarwal,

Milam A Brantley, Jr., Jonathan L Haines, William S Bush

**Table of Contents Page No.**

**Figures**

Figure S1. Genetic ancestry from principal component analysis. 2

Figure S2. Histogram of age, by case status. 3

Figure S3. Details of subsets of gene regions analyzed. 4

Figure S4. Number of overlapping SNPs between pathway pairs. 5

Figure S5. P-value for overlapping SNPs between pathway pairs. 6

Figure S6. Effect of smoking adjustment on pathway risk explained. 7

Figure S7. Risk explained from each pathway by AMD subtype. 7

**Tables**

Table S1. Weighted-by-age, expected population prevalence calculation. 8

Table S2. 19 known risk SNPs and nearby gene information. 9

Table S3. Risk explained excluding known risk SNPs and SNP in LD. 10

Table S4. SNP counts, PRE, and p-values for partitioned regions. 10

**Figure S1. Genetic ancestry from principal component analysis.**

**
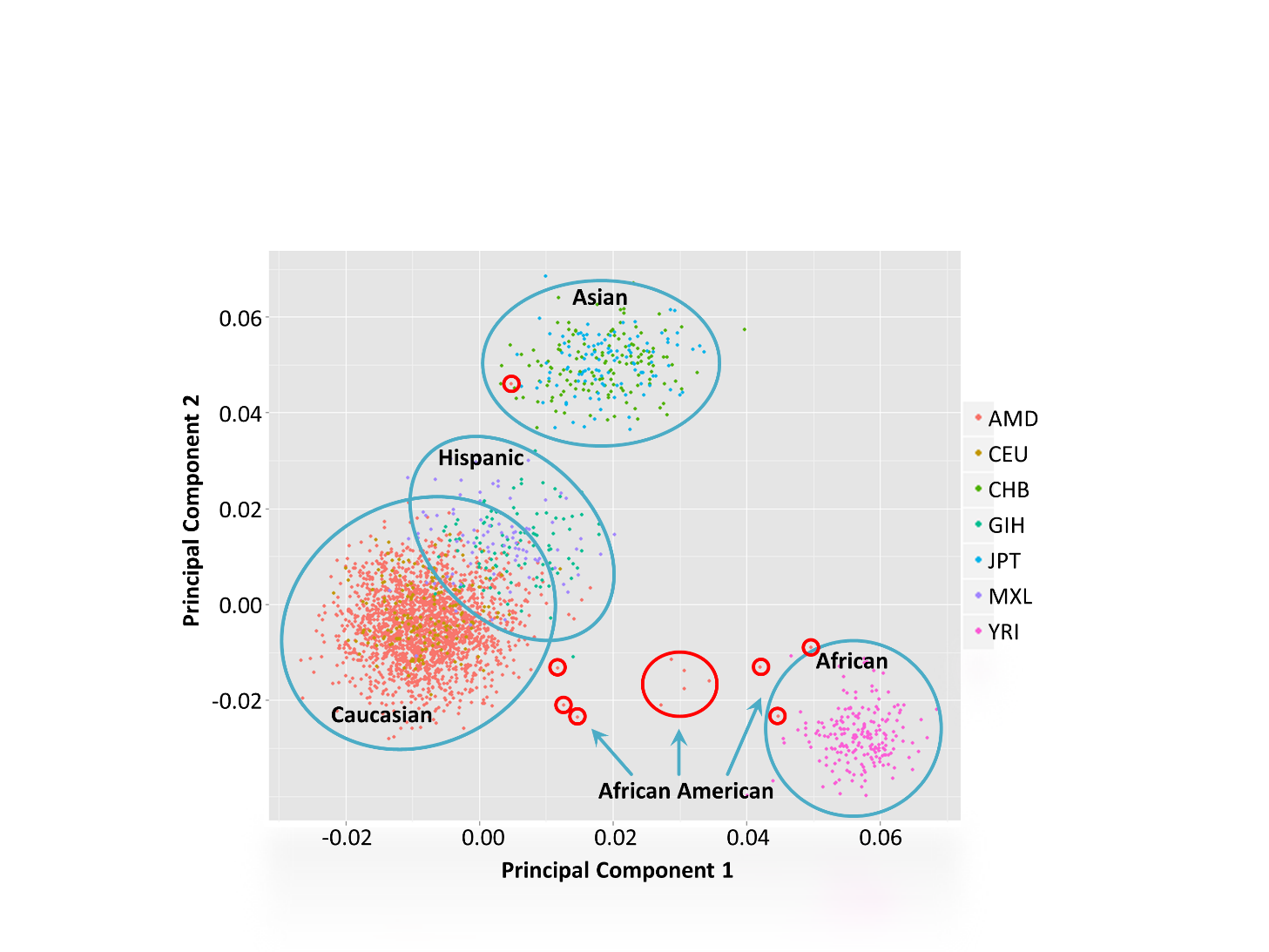
**

1,983 AMD dataset individuals (pre-QC)

805 HapMap individuals

- 165 CEU - Utah residents with Northern and Western European ancestry
- 137 CHB - Han Chinese in Beijing China
- 101 GIH - Gujarati Indians in Houston, Texas
- 113 JPT - Japanese in Tokyo, Japan
- 86 MXL - Mexican ancestry in Los Angeles, California
- 203 YRI - Yoruba in Ibadan, Nigeria

To calculate principal components, we used 71 ancestry-informative markers (AIMs) that were present in both HapMap and AMD individuals. 1 Asian-descent and 11 African American individuals were excluded from analysis (circled in red).

*Reference SNP (rs) numbers for 71 AIMs:*

rs3845596, rs2007350, rs1229133, rs1409778, rs2291409, rs6426327, rs520354, rs1868092, rs975612, rs972881, rs1521527, rs1435850, rs1320131, rs737516, rs1996818, rs1479371, rs1461131, rs1147696, rs2686085, rs225160, rs999634, rs736201, rs173686, rs1807912, rs1560550, rs31251, rs2296412, rs169125, rs942150, rs839556, rs369643, rs1080085, rs3294, rs901170, rs4107736, rs1440369, rs1868280, rs4246828, rs6474795, rs2151065, rs878400, rs7860423, rs11813505, rs722317, rs540819, rs236919, rs1630675, rs916041, rs1548837, rs903770, rs310935, rs1372177, rs981270, rs4904574, rs2873, rs1648282, rs1030588, rs936013, rs461785, rs168206, rs2164062, rs1019977, rs1426311, rs959419, rs1981431, rs186659, rs354731, rs816943, rs2837956, rs756658, rs739096

**Figure S2. Histogram of age, by case status.**

Age in years recorded at time of examination. Histogram for 668 controls and 1,145 cases. Individuals with no smoking status *not* excluded.

**Figure S3. Details of subsets of gene regions analyzed.**

*
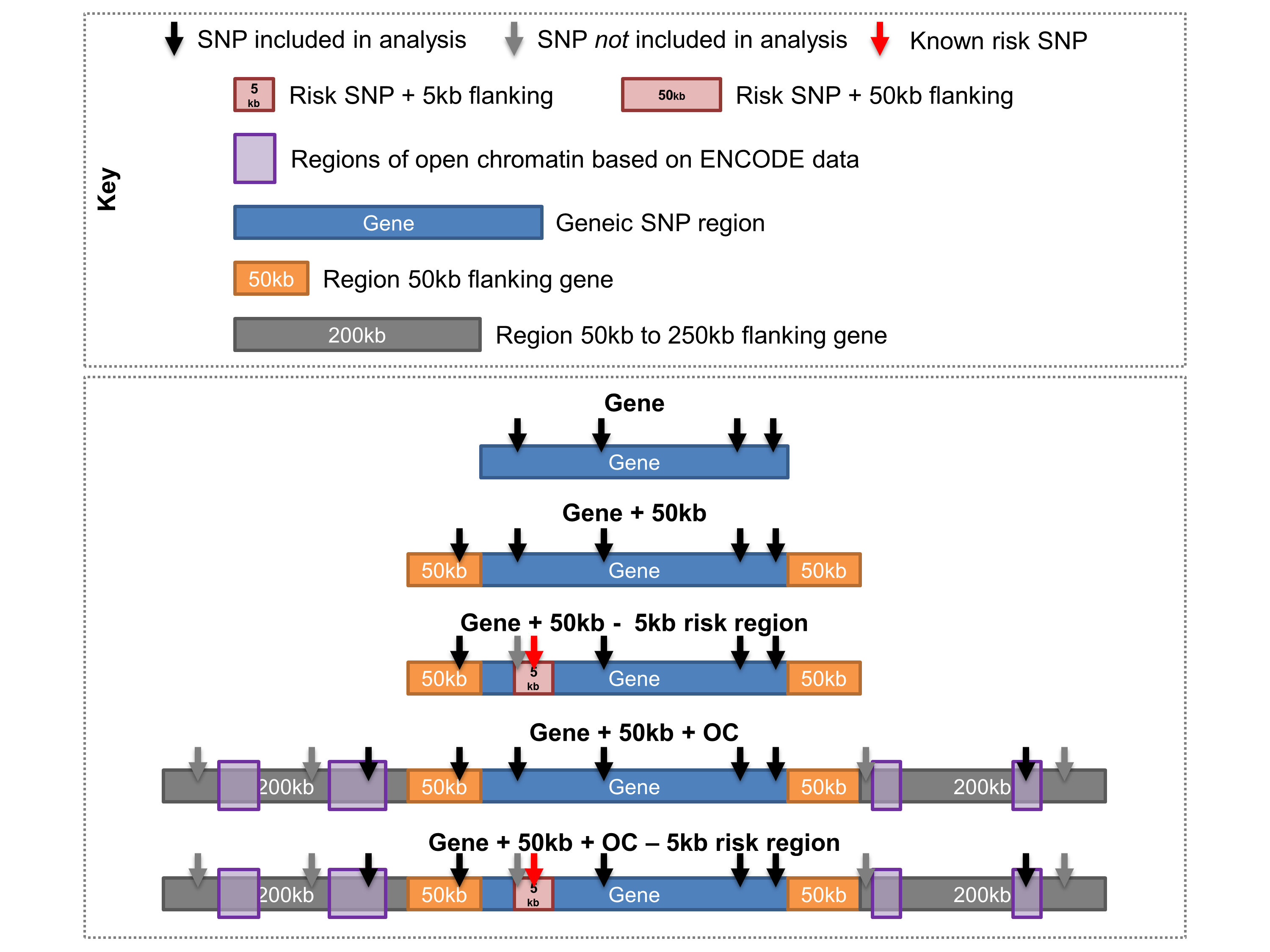
*

Open chromatin regions determined using narrow peak windows from ENCODE DNaseI hypersensitivity analyses in human RPE cells.

**Figure S4. Number of overlapping SNPs between pathway pairs.**

|  | Angiogenesis | Antioxidant | Apoptosis | Complement | Inflammatory | Nicotine | OxPhos |
| --- | --- | --- | --- | --- | --- | --- | --- |
| Antioxidant | 30 |  |  |  |  |  |  |
| Apoptosis | 6,542 | 583 |  |  |  |  |  |
| Complement | 316 | - | 349 |  |  |  |  |
| Inflammatory | 2,835 | 342 | 6,465 | 955 |  |  |  |
| Nicotine | 148 | 11 | 538 | - | 281 |  |  |
| OxPhos | 116 | 16 | 513 | - | 118 | - |  |
| TCA | 33 | - | 44 | - | 44 | - | 47 |

Pathway pairs with little or no overlapping SNPs shown as green, fading to red for pathway pairs with the most overlapping SNPs.

**Figure S5. P-value for overlapping SNPs between pathway pairs.**

|  | Angiogenesis | Antioxidant | Apoptosis | Complement | Inflammatory | Nicotine | OxPhos |
| --- | --- | --- | --- | --- | --- | --- | --- |
| Antioxidant | 0.5 |  |  |  |  |  |  |
| Apoptosis | 0.5 | 0.239 |  |  |  |  |  |
| Complement | 0.019 | - | 0.5 |  |  |  |  |
| Inflammatory | 0.5 | 0.049 | 0.294 | 1.11^-25^ |  |  |  |
| Nicotine | 0.130 | 0.127 | 0.491 | - | 0.380 |  |  |
| OxPhos | 0.116 | 0.5 | 0.5 | - | 0.5 | - |  |
| TCA | 0.5 | - | 0.5 | - | 0.5 | - | 0.221 |

Pathway pairs with overlapping SNPs contributing to smaller p-values shown as red, fading to green for pathway pairs with overlapping SNPs resulting large, non-significant p-values. Three pathway pairs shown have significant (< 0.05) p-values.

**Figure S6. Effect of smoking adjustment on pathway risk explained.**


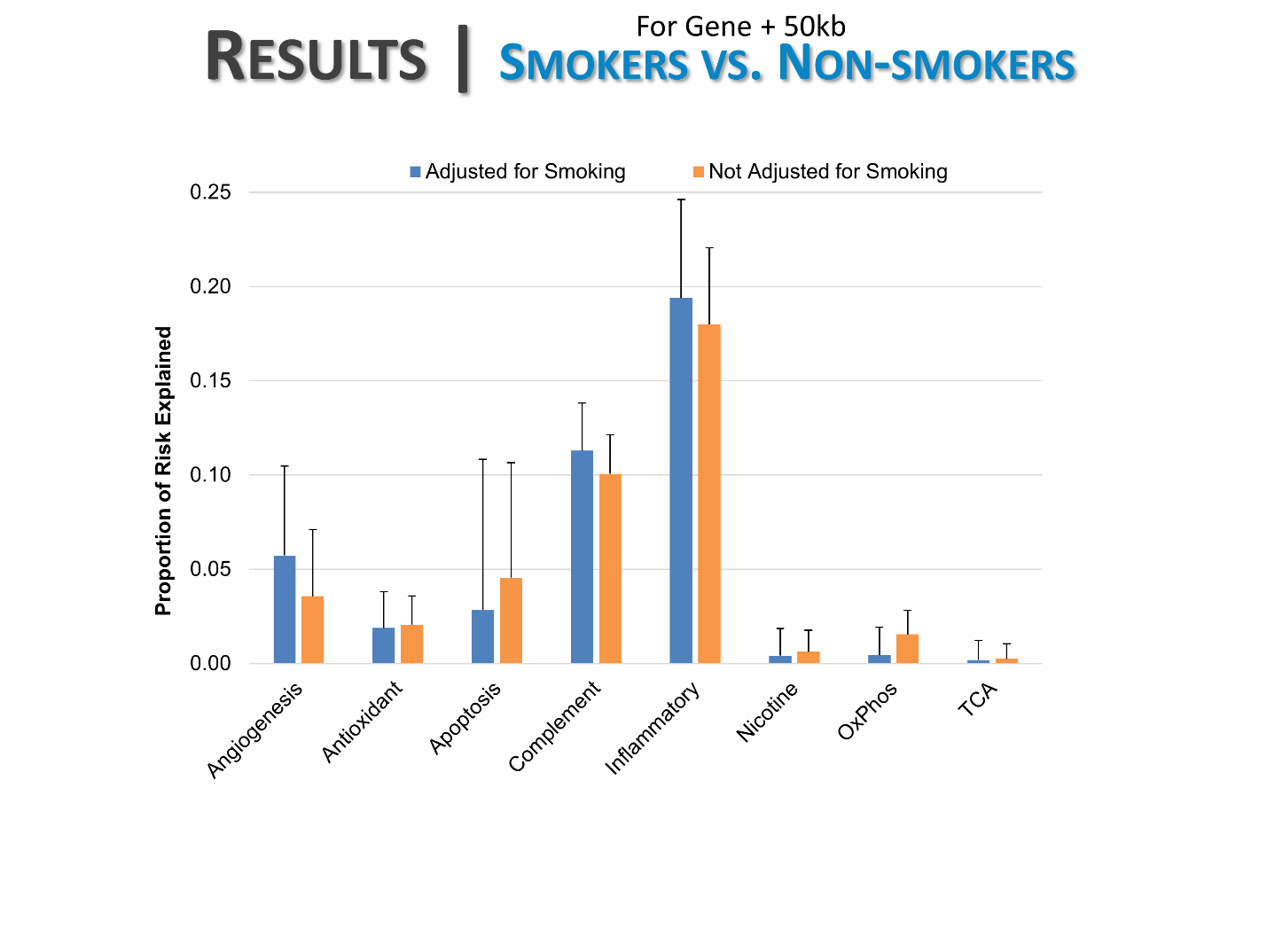


Gene plus 5kb flanking, minus risk plus 5kb flanking regions excluded.

**Figure S7. Risk explained from the angiogenesis pathway by AMD subtype.**


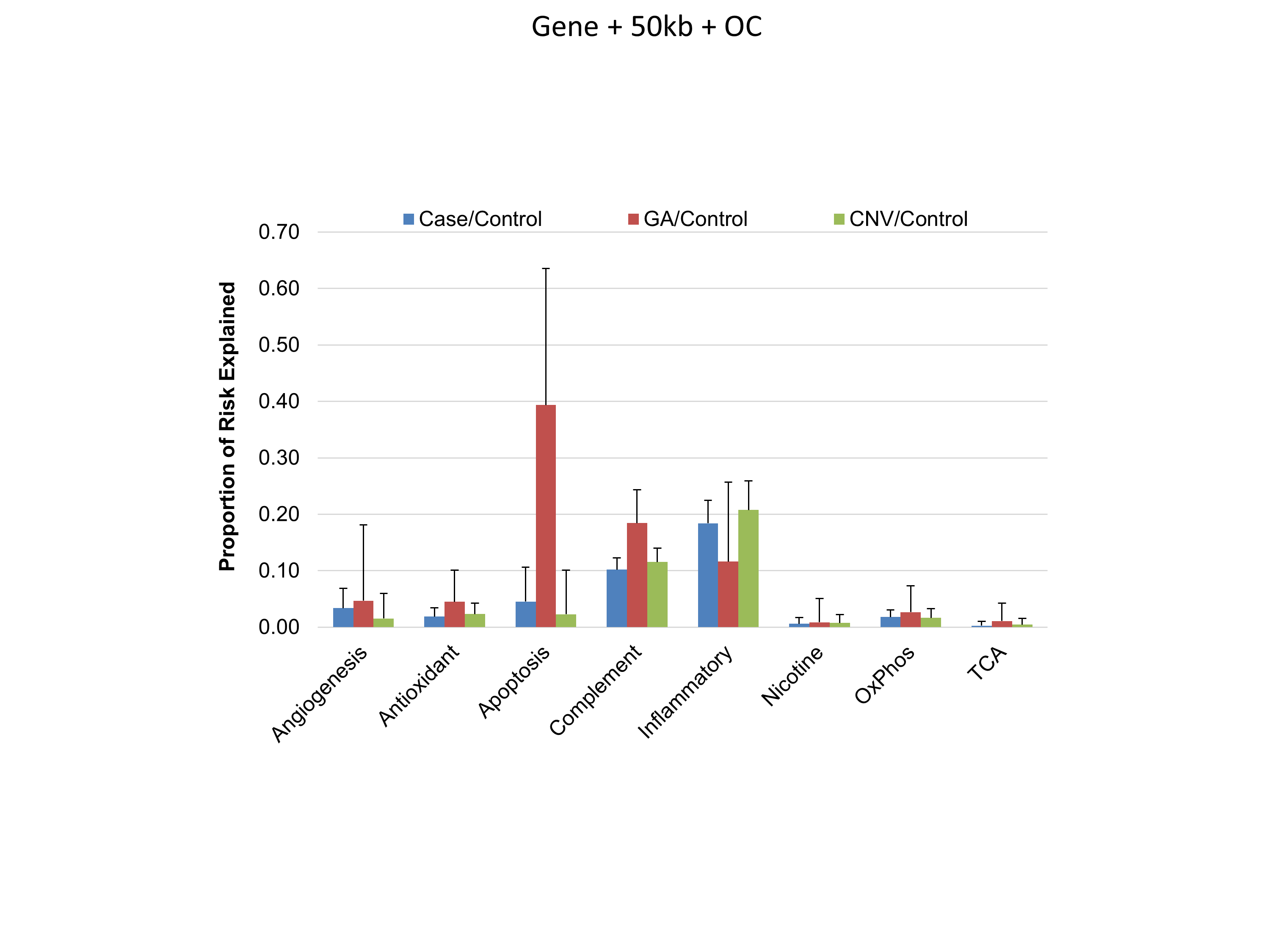


Genic SNPs plus 50kb plus open chromatin SNPs included in analyses. Standard error is very large for GA versus controls because of the low sample size of cases with GA in our dataset.

| Table S1. Weighted-by-age, expected population prevalence calculation. | | | |
| --- | --- | --- | --- |
| Age Range | **U.S. Prev.** | **Count** | **Count × Prev.** |
| 40-49 | 0.05 | 4 | 0.2 |
| 50-54 | 0.34 | 3 | 1.0 |
| 55-59 | 0.39 | 62 | 24.2 |
| 60-64 | 0.56 | 140 | 78.4 |
| 65-69 | 0.91 | 262 | 238.4 |
| 70-74 | 1.66 | 352 | 584.3 |
| 75-49 | 3.24 | 398 | 1289.5 |
| 80+ | 11.77 | 592 | 6967.8 |
| Total |  | **1813** | **9183.9** |

United States prevalence rates (percent of population for given age range) from Friedman et al. 2011 - PMID: 15078675.

[9183.9 / 1813] = 5.07% weighted, expected AMD prevalence rate

| **Table S2. 19 known risk SNPs and nearby gene information.** | | | | | |
| --- | --- | --- | --- | --- | --- |
| **RS Number** | **Chromosome** | **Position** | **Nearby Genes** | **Distance to index SNP (kb)** | **Location** |
| rs10490924 | 10 | 124214448 | *ARMS2* | 0 | Coding |
|  |  |  | *HTRA1* | 6.6 | Upstream |
| rs10737680 | 1 | 194946078 | *CFH* | 0 | Intronic |
| rs429608 | 6 | 32038441 | *C2* | 17 | Downstream |
|  |  |  | *CFB* | 10.6 | Downstream |
|  |  |  | *SKIV2L* | 0 | Intronic |
| rs2230199 | 19 | 6718387 | *C3* | 0 | Coding |
| rs5749482 | 22 | 31389665 | *TIMP3* | 137.1 | Upstream |
|  |  |  | *SYN3* | 0 | Intronic |
| rs4420638 | 19 | 45422946 | *APOE* | 10.3 | Downstream |
|  |  |  | *APOC1* | 5 | Downstream |
| rs1864163 | 16 | 55554734 | *CETP* | 0 | Intronic |
| rs943080 | 6 | 43934605 | *VEGFA* | 72.4 | Downstream |
| rs13278062 | 8 | 23082971 | *TNFRSF10A* | 0.3 | Upstream |
| rs920915 | 15 | 58688467 | *LIPC* | 35.7 | Upstream |
| rs4698775 | 4 | 110590479 | *CFI* | 71.4 | Downstream |
|  |  |  | *CCDC109B* | 0 | Intronic |
| rs3812111 | 6 | 116443735 | *COL10A1* | 0 | Intronic |
| rs13081855 | 3 | 99481539 | *COL8A1* | 0 | Intronic |
| rs3130783 | 6 | 30774357 | *IER3* | 62 | Upstream |
|  |  |  | *DDR1* | 77.5 | Upstream |
| rs8135665 | 22 | 38476276 | *SLC16A8* | 0 | Intronic |
| rs334353 | 9 | 100948186 | *TGFBR1* | 0 | Intronic |
| rs8017304 | 14 | 68785077 | *RAD51B* | 0 | Intronic |
| rs6795735 | 3 | 64705365 | *ADAMTS9* | 32 | Upstream |
|  |  |  | *ADAMTS9-AS2* | 0 | Intronic |
|  |  |  | *MIR548A2* | 0.3 | Upstream |
| rs9542236 | 13 | 30717325 | *B3GALTL* | 0 | Intronic |
| Adapted from Fritsche 2013 (doi:10.1038/ng.2578). | | | |  |  |

| **Table S3. Risk explained excluding known risk SNPs and SNP in LD.** | | | | |
| --- | --- | --- | --- | --- |
| **Exclusion Criteria** | **Number of SNPs Excluded** | **PVE (%)** | **SE (%)** | **p-val.** |
| None | 0 | 36.72 | 16.13 | 0.0042 |
| R^2^ ≥ 10% | 1,183 | 35.25 | 14.75 | 0.0063 |
| R^2^ ≥ 5% | 1,684 | 35.19 | 14.75 | 0.0064 |
| R^2^ ≥ 1% | 1,925 | 35.12 | 14.75 | 0.0064 |
| 1MB flanking | 9,938 | 31.33 | 14.70 | 0.0145 |
| 19 known risk SNPs: PVE ~13.3%, SE ~3.92%, p-val. < 1.35×10^-61^ for all  Remainder SNPs with none excluded: 659,162  PVE, SE, and p-values shown for non-known risk SNPs, excluding specified SNPs  R^2^: 0 = linkage equilibrium ; 1 = perfect linkage disequilibrium | | | | |

| Table S4. SNP counts, proportion of risk explained, and p-values for partitioned regions. | | | | | |
| --- | --- | --- | --- | --- | --- |
|  | **GO Term Pathway** | **G** | **G+50** | **G+50+OC** | **G+50+OC**  **minus R+5** |
| Number of SNP | Angiogenesis | 8,853 | 16,907 | 17,465 | 17,455 |
|  | Antioxidant Activity | 616 | 2,014 | 2,115 | 2,114 |
|  | Apoptotic Signaling | 26,682 | 54,417 | 56,136 | 56,130 |
|  | Complement Activation | 478 | 1,343 | 1,374 | 1,370 |
|  | Inflammatory Response | 5,883 | 15,993 | 16,635 | 16,630 |
|  | Response to Nicotine | 426 | 1,101 | 1,150 | 1,150 |
|  | Oxidative Phosphorylation | 350 | 1,451 | 1,548 | 1,548 |
|  | Tricarboxylic Acid Cycle | 342 | 981 | 1,019 | 1,019 |
| Proportion of Risk Explained | Angiogenesis | 0.0427 | 0.0356 | 0.0337 | 0.0309 |
|  | Antioxidant Activity | 0.0059 | 0.0205 | 0.0189 | 0.0178 |
|  | Apoptotic Signaling | 0.0405 | 0.0454 | 0.0454 | 0.0438 |
|  | Complement Activation | 0.0715 | 0.1006 | 0.1020 | 0.0976 |
|  | Inflammatory Response | 0.1346 | 0.1799 | 0.1839 | 0.1786 |
|  | Response to Nicotine | 0.0020 | 0.0063 | 0.0060 | 0.0060 |
|  | Oxidative Phosphorylation | 0.0093 | 0.0154 | 0.0179 | 0.0179 |
|  | Tricarboxylic Acid Cycle | 0.0057 | 0.0024 | 0.0026 | 0.0026 |
| P-value | Angiogenesis | 0.0797 | 0.3017 | 0.3592 | 0.4410 |
|  | Antioxidant Activity | 0.5000 | 0.1081 | 0.1439 | 0.1631 |
|  | Apoptotic Signaling | 0.2828 | 0.5000 | 0.5000 | 0.5000 |
|  | Complement Activation | 1.2E-28 | 7.1E-27 | 6.3E-27 | 6.8E-26 |
|  | Inflammatory Response | 6.3E-11 | 3.1E-08 | 4.4E-08 | 9.5E-08 |
|  | Response to Nicotine | 0.5000 | 0.5000 | 0.5000 | 0.5000 |
|  | Oxidative Phosphorylation | 0.2232 | 0.1103 | 0.0802 | 0.0804 |
|  | Tricarboxylic Acid Cycle | 0.4602 | 0.5000 | 0.5000 | 0.5000 |

G=Gene; 50=50kb flanking gene; OC=SNPs in open chromatin 50kb to 250kb flanking; R+5=Risk SNPs plus 5kb flanking
